# The effect of vitamin D deficiency during pregnancy on adverse birth outcomes in neonates: a systematic review and meta-analysis

**DOI:** 10.3389/fped.2024.1399615

**Published:** 2024-05-14

**Authors:** Zhiying You, Hua Mei, Yayu Zhang, Dan Song, Yanbo Zhang, Chunli Liu

**Affiliations:** Department of Neonatology, Affiliated Hospital of Inner Mongolia Medical University, Huhhot, China

**Keywords:** vitamin D deficiency, pregnancy, adverse birth outcomes, neonate, preterm, LBWI, SGA, meta-analysis

## Abstract

**Objective:**

To systematically evaluate the effect of vitamin D deficiency during pregnancy on neonatal adverse outcomes, such as preterm infants, low birth weight infants (LBWI), and small for gestational age (SGA) infants.

**Methods:**

A comprehensive literature search was conducted across multiple databases including PubMed, Embase, Cochrane Library, SinoMed, Wanfang Data Knowledge Service Platform, China National Knowledge Internet (CNKI), and VIP Chinese Science and Technology Journal Database (VIP). Following predefined inclusion and exclusion criteria, two researchers independently screened, extracted data, and assessed the quality of the included studies. Meta-analysis was performed using RevMan 5.4 and Stata 14 software to synthesize the findings.

**Results:**

This study incorporated 13 cohort studies from 8 different countries and regions, encompassing a total of 55,162 pregnant women, among whom 28,155 were identified as having vitamin D deficiency. The Newcastle-Ottawa Scale (NOS) score ranged from 7–9 points. Meta-analysis results indicated a higher incidence of LBWI (OR = 5.52, 95% CI = 1.31–23.22. *P = *0.02) in the group of pregnant women with vitamin D deficiency compared to those with adequate levels. However, there was no statistically significant difference in the likelihood of premature birth (OR = 1.25, 95% CI = 0.78–1.99. *P *= 0.36) or SGA (OR = 1.47, 95% CI = 0.81–2.68. *P *= 0.21) among newborns born to mothers with vitamin D deficiency vs. those with sufficient levels of vitamin D. Subgroup analysis based on the timing of maternal blood collection revealed that there was no statistically significant association between vitamin D levels during pregnancy and the incidence of preterm birth across all stages of pregnancy. Furthermore, vitamin D deficiency throughout the entire pregnancy was associated with an increased incidence of neonatal LBWI, whereas vitamin D levels during the first, second, and third trimesters did not demonstrate statistically differences on LBWI. Neonates born to mothers with vitamin D deficiency throughout pregnancy were found to have a higher likelihood of developing SGA. However, there was no statistically significant association between vitamin D levels and the development of SGA during the first and second trimesters.

**Conclusions:**

Adequate levels of vitamin D during pregnancy may decrease the incidence of LBWI, although further research is needed to determine its impact on the occurrence of preterm birth and SGA.

**Systematic Review Registration:**

https://www.crd.york.ac.uk/prospero/display_record.php?ID=CRD42024535950, **Identifier:** (CRD42024535950).

## 1 Introduction

Adverse birth outcomes in neonates primarily encompass preterm birth, low birth weight infants (LBWI), and small for gestational age (SGA) infants. Preterm birth is characterized by neonates born before 37 weeks gestation, while SGA refers to infants with a birth weight below the 10th percentile for their gestational age. LBWI are infants born weighing less than 2,500 grams. In recent years, there has been a noticeable rise in the prevalence of neonates adverse outcomes, attributed to various factors such as maternal pregnancy complications, inadequate dietary habits, lifestyle choices, socioeconomic status, environmental factors, and others (1, 2). According to statistical data, the global incidence of preterm birth in 2019 was reported at 10.6% (with a range of 9.0%–12.0%), while in China, the incidence was 6.9% (with a range of 5.8%–7.9%) (3). Research has indicated that the prevalence of SGA infants in China ranges from 6.05%–13.1% (4). Adverse birth outcomes in neonates are a significant contributor to neonatal mortality rates and increase the likelihood of developing chronic noninfectious diseases in adulthood (5, 6). Therefore, mitigating the risk of morbidity and enhancing the intrauterine fetal microenvironment during gestation are crucial strategies for improving neonatal survival and long-term quality of life.

Vitamin D is a crucial micronutrient that plays a significant role in influencing pregnancy outcomes and fetal development through its regulation of hormone secretion, cellular proliferation, and differentiation. Given the unique physiological changes experienced by pregnant women, including heightened levels of estrogen and progesterone and an increased need for Vitamin D, the prevalence of Vitamin D deficiency as a concurrent condition during pregnancy has risen in recent times (7, 8). Several studies have indicated that approximately 50% of the global population is susceptible to Vitamin D deficiency and insufficiency (9). Specifically, the rates of Vitamin D deficiency among urban and rural pregnant women in China are reported to be 74.3% and 38.7%, respectively (10, 11). Vitamin D deficiency during pregnancy has been linked to various adverse maternal outcomes including gestational diabetes, eclampsia, and miscarriage. However, the impact on preterm labor, LBWI, and SGA infants remains a topic of debate. This study employs systematic review and meta-analysis techniques to investigate the effects of Vitamin D deficiency during pregnancy on prematurity, LBWI, SGA infants, with the aim of mitigating negative neonatal birth outcomes and establishing a foundation for early clinical intervention.

## 2 Methods

### 2.1 Literature inclusion and exclusion criteria

The inclusion criteria for this study include: the protocol for this review was recorded in the International Prospective Register of Systematic Reviews (PROSPERO) with the ID CRD42024535950. The research adheres to the guidelines outlined in the Preferred Reporting Items for Systematic reviews and Meta-Analyses (PRISMA) statement (12). The search strategy was based on the PICOS (P: Population; I: Intervention; C: Comparison; O: Outcome; S: Study design) methodology. The PICOS framework is as follows: Population—singleton pregnant women; Intervention—vitamin D deficiency (25-OH-vitD3 < 20 ng/ml) (9); Comparison—adequate levels of vitamin D (25-OH-vitD3 ≥ 30 ng/ml) (9) in the control group; Outcome—occurrence of preterm labor (<37 weeks' gestation), LBWI (less than 2,500 g), or SGA (birth weight less than 10th percentile for gestational age) (13); and Study design—cohort or case-control study.

The exclusion criteria for this study include: (1) incomplete data where the full text is unavailable; (2) conference abstracts, systematic evaluations, reviews, case reports of animal experiments, expert consensus, and master's and doctoral degree papers; (3) duplicated publications; (4) literature not in Chinese or English; and (5) low-quality literature with questionable data authenticity.

### 2.2 Literature search

A systematic review was conducted to examine the impact of vitamin D deficiency during pregnancy on preterm delivery, low birth weight infants, and small for gestational age infants. Literature searches were conducted in databases including PubMed, Embase, Web of Science, Cochrane Library, VIP Chinese Science and Technology Journal Database (VIP), China National Knowledge Internet (CNKI), Wanfang Database, and Sinomed, with search terms in both Chinese and English languages. The search strategy employed for this meta-analysis included keywords such as “Pregnancy” OR “Pregnancies” OR “Gestation” AND “Vitamin D Deficiency” OR “Vitamin D Deficiencies” OR “Deficiencies, Vitamin D” AND “newborn” OR “infants” OR “neonate” up until August 2023. A combination search approach utilizing MeSH subject terms and free terms was employed, with references being systematically traced back to the included literature. The detailed search strategy for PubMed is outlined in Supplementary Table S1.

### 2.3 Data extraction

Two researchers (You ZY and Zhang YY) conducted independent screening of the literature for data extraction, cross-checking their findings. Any discrepancies were resolved through consensus discussion or consultation with a third party. The data extraction process encompassed: basic information of the included studies, including study title, first author, year of publication, study type, baseline characteristics, diagnostic criteria of study subjects, risk of bias evaluation, outcome indicators, and outcome measures of interest.

### 2.4 Quality evaluation

The quality of the literature was evaluated using the Newcastle-Ottawa Scale (NOS) for cohort and case-control studies. The evaluation of cohort studies encompassed three primary dimensions: selection of study population, comparability between study groups, and measurement of outcomes, with scores on the NOS ranging from 0–9 points. Two researchers (You ZY and Zhang YY) independently conducted assessments, with a third researcher available to resolve any discrepancies. Studies receiving a score of 7–9 were deemed to be of high quality, indicating a low risk of bias.

### 2.5 Statistical analysis

A meta-analysis was conducted using RevMan54 and Stata 14 software to systematically assess the impact of vitamin D deficiency during pregnancy on adverse birth outcomes, specifically preterm infants, LBWI, and SGA infants. Mean ± standard deviation (MD) was utilized as the effect indicator for continuous data, while odds ratio (OR) was used for categorical data, with each effect size reported with a 95% confidence interval (CI). Heterogeneity was assessed through the application of the Chi-square test and *I^2^* statistic. When *I^2 ^*> 50% and *P *≤ 0.1, it signified heterogeneity among the studies. In such cases, the random-effects model was employed to compute the combined statistic, and subgroup or sensitivity analyses were conducted to explore the underlying sources of heterogeneity. Conversely, if these criteria were not met, the fixed-effects model was utilized. Additionally, funnel plots were generated for the outcome measures, and publication bias was evaluated through the integration of Egger's and Begg's tests. There was significant difference between the two group when *P *≤ 0.05.

## 3 Results

### 3.1 Results of the literature search and basic characteristics of the included studies

**Figure 1:**
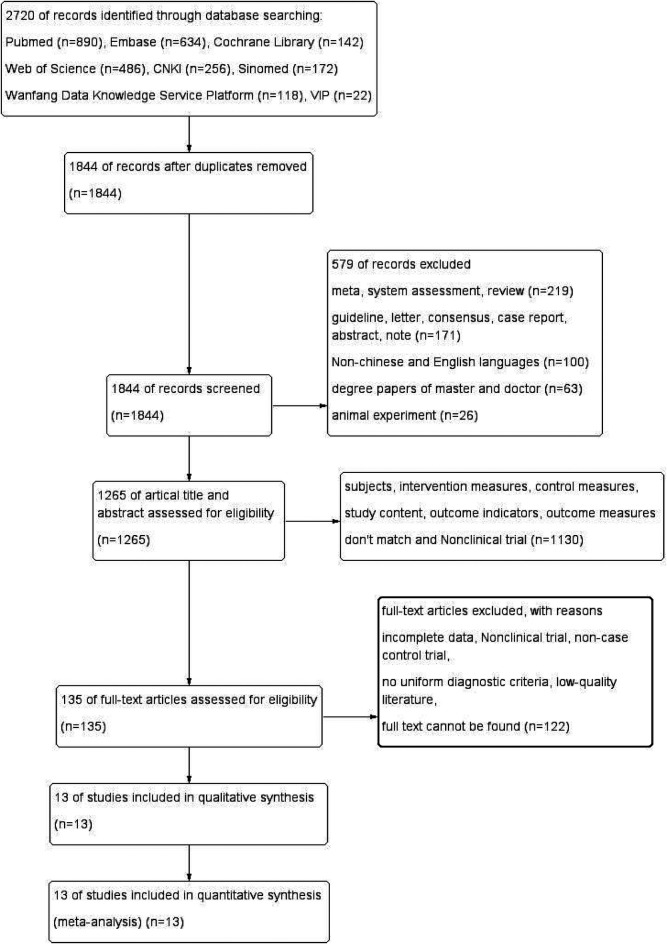
PRISMA flow diagram of study selection.

A total of 2,720 articles were obtained, comprising 568 articles in Chinese (256 articles in CNKI, 118 articles in Wanfang database, 22 articles in VIP database, and 172 articles in Sinomed) and 2,152 articles in English (890 articles in PubMed, 634 articles in Embase, 142 articles in Cochrane library, 486 articles in Web of Science). 876 duplicates were removed, and 579 articles were excluded due to their meta, systematic evaluation, case report, expert consensus, animal test, etc. After reviewing titles, and abstracts, a total of 1,130 articles were excluded for non-compliance with intervention and control measures, study content, and outcome indicators. After reviewing full texts, an additional 122 articles were excluded for reasons such as inability to locate full text, incomplete data, non-case-control or cohort study design, and low quality. Ultimately, 13 cohort studies (8, 14–25) were included in the literature screening process, as depicted in Figure 1. The 13 publications were published from 2012–2021, covering a total of 8 countries and regions, involving 28,155 cases in the group with vitamin D deficiency in pregnancy (experimental group) and 8,580 cases in the group with adequate levels of vitamin D (control group). The basic information of the literature is shown in Supplementary Table S2.

### 3.2 Literature quality assessment

Thirteen cohort studies (8, 14–25) were assessed for quality using the NOS, with two articles receiving a score of 7 (18, 25), seven articles receiving a score of 8 (14, 16, 17, 19, 21–23), and four articles receiving a score of 9 (8, 15, 20, 24), indicating high-quality literature. The definitions of vitamin D deficiency in pregnancy and gestational staging in these studies were based on standardized criteria. The quality scores of the included articles can be found in Supplementary Table S3.

### 3.3 Meta-analysis

**Figure 2:**
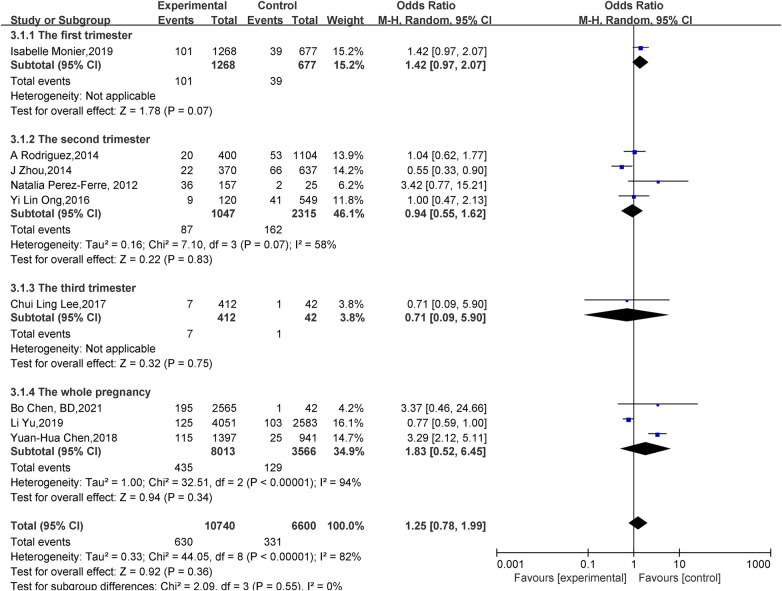
Forest plot for preterm.

Effect of vitamin D deficiency in pregnancy on preterm infants:A total of nine papers (8, 14, 15, 17–20, 22, 23) reported the effect of vitamin D deficiency in pregnancy on preterm labor. Statistical heterogeneity existed among the studies (*P *< 0.00001, *I^2 ^*= 82%). Meta-analysis was performed using a random-effects model, which showed that in the vitamin D deficiency group during pregnancy, compared with the adequacy group, the Preterm birth rate was not statistically significant (OR = 1.25, 95% CI 0.78–1.99, *P* = 0.36); subgroup analysis was performed according to the gestational week of maternal vitamin level measurement: there was no statistically significant effect of vitamin D deficiency on the occurrence of preterm birth in the first (OR = 1.42, 95% CI 0.97–2.07, *P *= 0.07), second (OR = 0.94, 95% CI 0.55–1.62, *P *= 0.83), third (OR = 0.71, 95% CI 0.09–5.90, *P *= 0.75) trimester of pregnancy, and the whole (OR = 1.83, 95% CI 0.52–6.45, *P *= 0.34) pregnancy (Figure 2).

**Figure 3:**
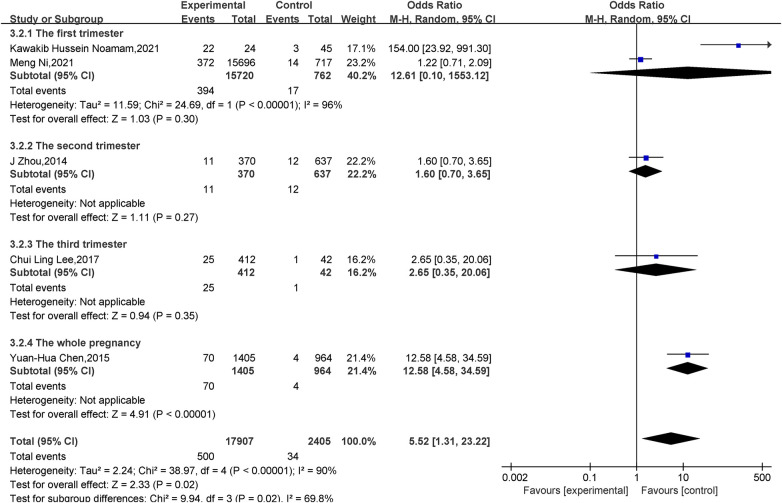
Forest plot for LBWI.

Effect of vitamin D deficiency in pregnancy on LBWI: A total of five papers (14, 16, 18, 21, 25) reported the effect of vitamin D deficiency in pregnancy on LBWI, and there was statistical heterogeneity among the studies (*P *< 0.001, *I^2 ^*= 90%). Meta-analysis was performed using a random-effects model, and the results showed that neonates in the deficient group were more likely to develop LBWI than those in the vitamin adequate group during pregnancy, and the difference was statistically significant(OR = 5.52, 95% CI = 1.31–23.22, *P = *0.02); subgroup analyses were performed according to the gestational week in which the vitamin levels of pregnant mothers were measured: the whole pregnancy (OR = 12.58, 95% CI = 4.58–34.59, *P *< 0.001) found that vitamin D level deficiency increased the incidence of LBWI, while there was no statistically significant difference in the effect of vitamin D levels on LBWI in the first (OR = 12.61, 95% CI = 0.10–1,553.12, *P *= 0.30), second (OR = 1.60, 95% CI = 0.70–3.65, *P *= 0.27) and third (OR = 2.65, 95% CI = 0.35–20.60, *P *= 0.35) trimesters (Figure 3).

**Figure 4:**
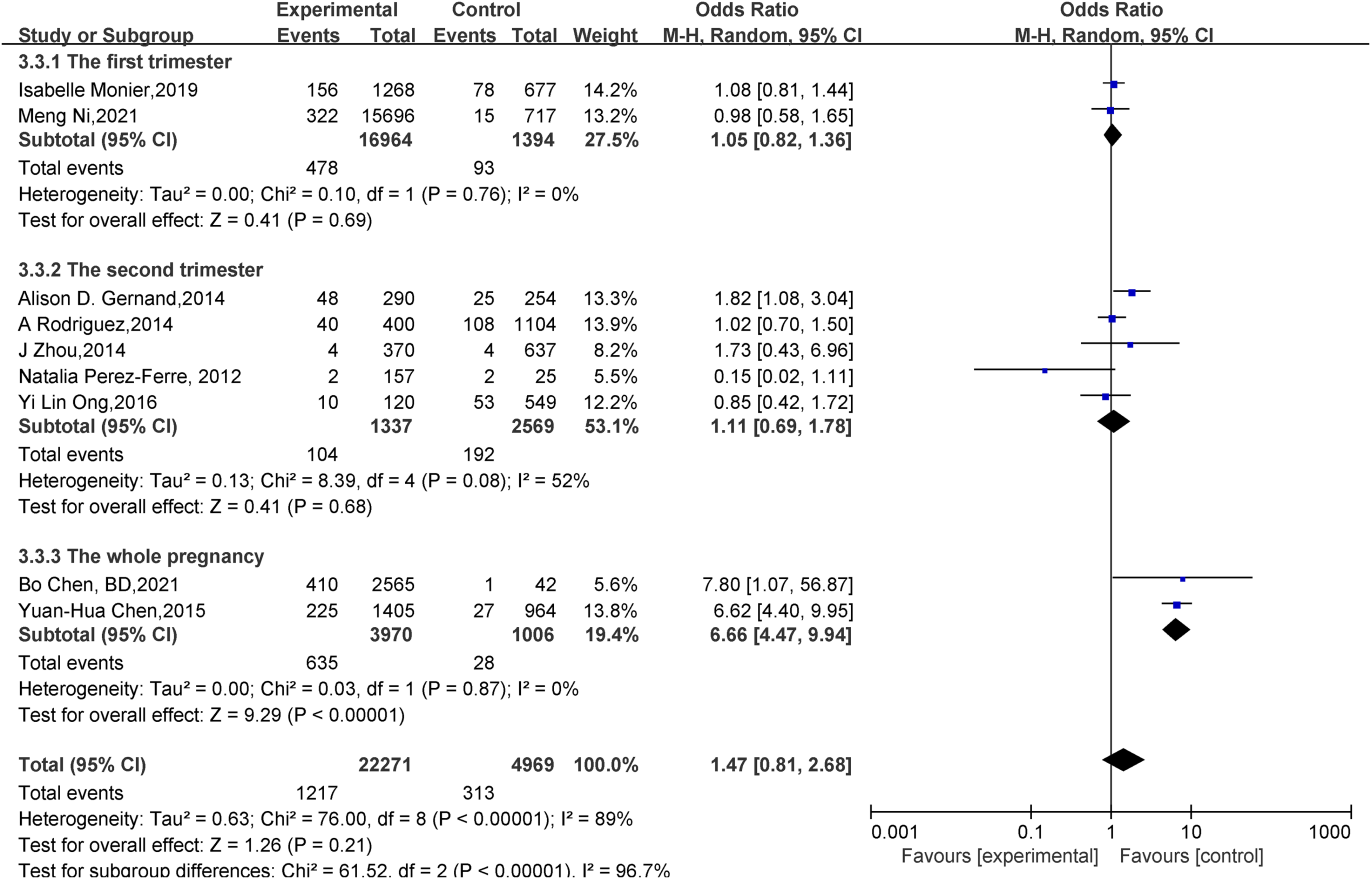
Forest plot for SGA.

Effect of vitamin D deficiency in pregnancy on SGA: Nine studies (14–17, 20–24) reported the effect of vitamin D deficiency in pregnancy on SGA, and there was statistical heterogeneity among studies (*P *< 0.001, *I^2 ^*= 89%), a random-effects model was used for meta-analysis, and the results showed that there was no statistically significant difference in the incidence of SGA in newborns between the vitamin D deficiency group and the adequate group during pregnancy (OR = 1.47, 95% CI = 0.81–2.68, *P *= 0.21). Subgroup analyses were conducted according to vitamin D deficiency included in gestational age: neonates with vitamin D deficiency were more likely to develop SGA throughout pregnancy (OR = 6.66, 95% CI = 4.47–9.94, *P* < 0.001), and the effect of vitamin D levels on SGA in the first (OR = 1.05, 95% CI = 0.82–1.36, *P *= 0.69) and second (OR = 1.11, 95% CI = 0.69–1.78, *P *= 0.68) trimesters was statistically insignificant (Figure 4).

### 3.4 Publication bias and sensitivity analysis

**Figure 5:**
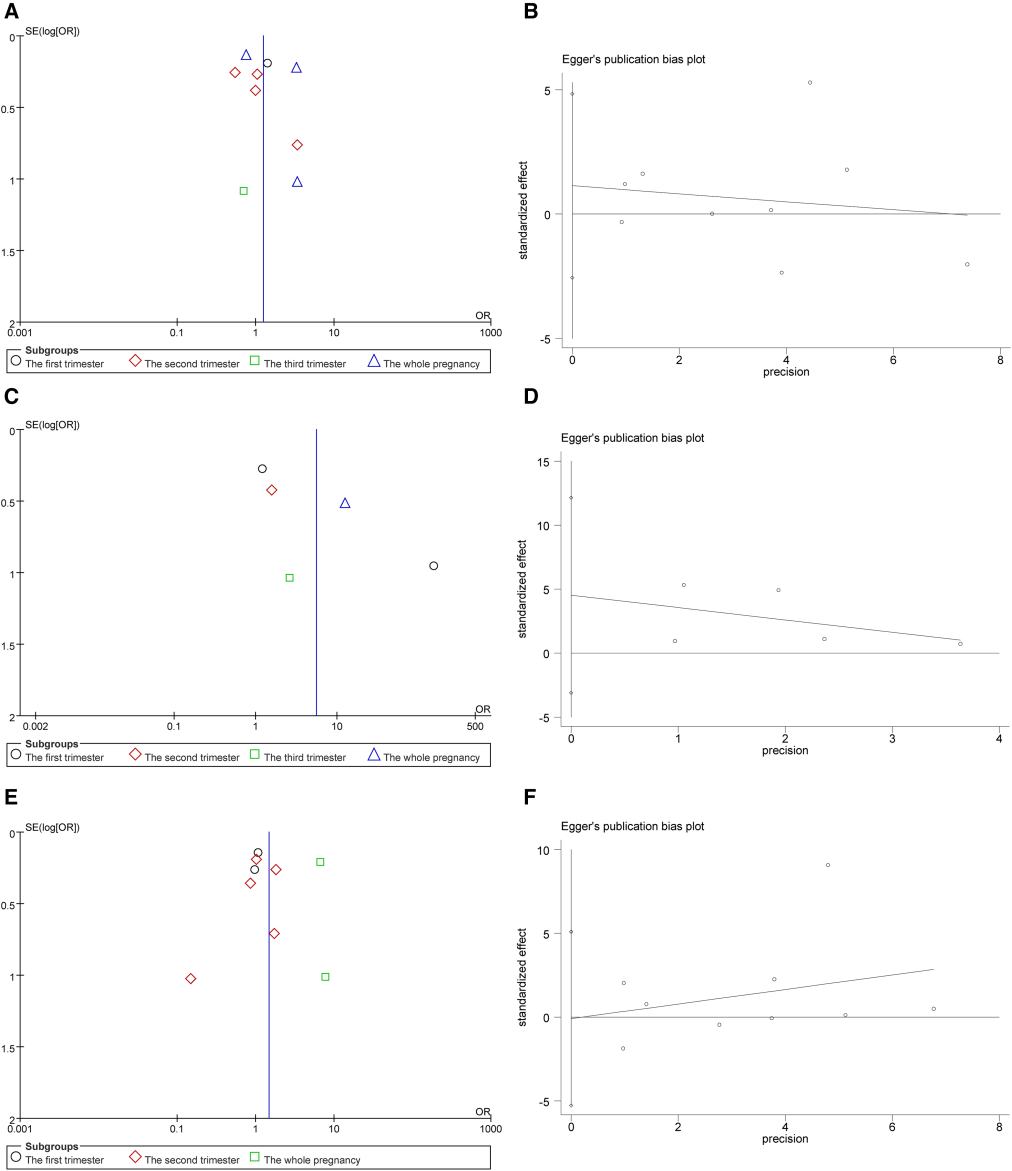
(**A**) Funnel plot for the premature infant. (**B**) Preterm publication bias egger test. (**C**) Funnel plot for LBWI. (**D**) LBWI publication bias egger test. (**E**) Funnel plot for SGA. (**F**) SGA publication bias egger test.

**Figure 6:**
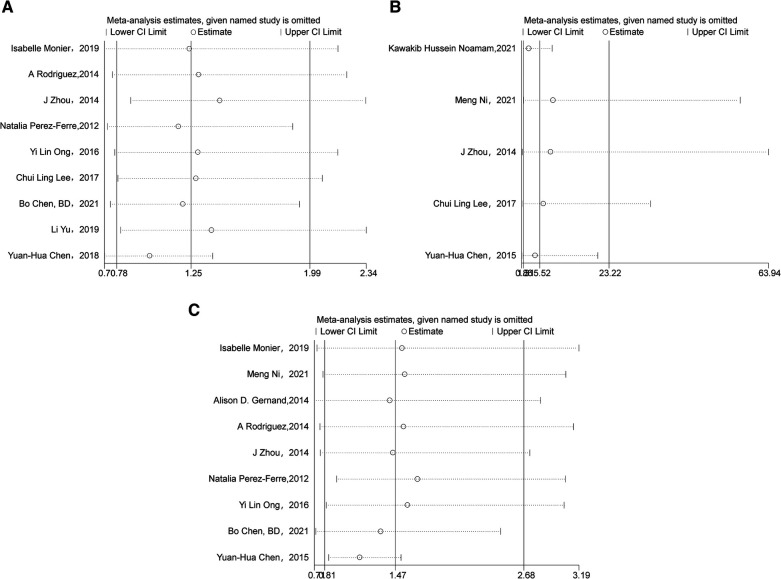
(**A**) Sensitivity analysis of preterm. (**B**) Sensitivity analysis of LBWI. (**C**) Sensitivity analysis of SGA.

We use inverted funnel plots analysis of each outcome to evaluate the publication bias among the studies (Figures 5A,C,E). The results showed that the dots in the funnel plot were basically symmetrical, indicating that the possibility of publication bias was small. Egger test results showed that no significant publication bias was found (all *P *> 0.05) (Figures 5B,D,F). Sensitivity analyses were conducted on birth outcomes. Deleting individual studies had minimal impact on the overall results, indicating the differences between studies are small and the analysis is highly reliable (Figures 6A–C).

## 4 Discussions

Preterm infants, LBWI, and SGA neonates are prevalent adverse birth outcomes that can lead to substantial and enduring medical and financial challenges for newborns and their families. While various factors are implicated in the occurrence of adverse neonatal birth outcomes, the precise etiology remains uncertain. Vitamin D deficiency in pregnant women is a common occurrence, attributed to heightened vitamin D needs, lifestyle modifications during pregnancy, reduced outdoor exposure, insufficient focus on and timely administration of vitamin D supplementation, as well as alterations in hormonal levels and metabolic status (26). The correlation between maternal vitamin D deficiency during pregnancy and adverse neonatal birth outcomes, including preterm infants, LBWI, and SGA infants, has garnered significant interest among researchers. Studies suggest that this association may be attributed to the inflammatory response during pregnancy and the modulation of the immune system by vitamin D. Pregnancy induces alterations in the placental meconium environment and cytokine regulatory network, potentially causing dysregulation of local inflammatory processes that may compromise placental function and result in adverse outcomes such as preterm birth, LBWI, SGA infants, and other complications. The impact of vitamin D levels on preterm infants may be attributed to various mechanisms. Specifically, in pregnant women with co-infectious diseases, vitamin D has been shown to decrease the production of inflammatory cytokines. Conversely, vitamin D deficiency may impede the inhibition of inflammation, potentially leading to chorioamnionitis infection and an elevated risk of preterm labor (27). These findings underscore the significance of vitamin D in modulating the anti-inflammatory pathway through the inhibition of nuclear factor-kB, ultimately contributing to the prevention of preterm birth (28). Furthermore, polymorphisms in the vitamin D receptor genes Fok-1 and Cdx-2 have been identified as potential contributors to the onset of preterm labor (29). Additionally, vitamin D has been shown to mitigate the risk of preterm labor by modulating the activity of adrenocorticotropic hormone-releasing hormone and other labor-related mediators in human syncytiotrophoblast cells (30). Sufficient maternal levels of vitamin D have been linked to a decreased incidence of LBWI and SGA infants, impacting fetal growth and development through the regulation of calcium homeostasis and parathyroid hormone levels (31, 32). This Meta-analysis incorporated 13 cohort studies, all of which received high quality scores. The findings of the Meta-analysis indicated that there was no significant difference in the incidence of preterm birth among newborns born to pregnant mothers in the vitamin D deficient group compared to those in the adequacy group, consistent with previous literature conclusions (15, 17–22). The findings of previous studies in the literature (14, 15, 17, 18, 20, 21, 23) suggest that there is no significant correlation between maternal vitamin D levels during pregnancy and the occurrence of SGA infants, which aligns with the results of the current study. However, for the outcome of low birth weight infants (LBWI), the incidence was greater in the group with vitamin D deficiency during pregnancy, consistent with existing literature (16, 22, 24, 26) but conflicting with the findings of A Rodriguez et al. (14, 15, 17, 18, 20, 21).

## 5 Limitations

This study is subject to several limitations. Firstly, the literature included in the analysis was restricted to Chinese and English sources, and despite efforts to limit the sample size of the included studies, some still had smaller sample sizes. Additionally, there were variations in the study factors selected across different studies, including gestational week, age, and race, which may introduce selection bias. As a result, it is imperative to confirm the findings of this study through further research with higher quality and larger sample sizes.

## 6 Conclusions

In summary, pregnant women who experience a deficiency in combined vitamin D levels during pregnancy may face a heightened risk of LBWI, while the likelihood of preterm birth and SGA remains unaffected. Given the limited sample size of the study, further research with a larger sample size and a multicenter approach is warranted to elucidate the impact of combined vitamin D levels during pregnancy on adverse birth outcomes. Consequently, regular monitoring of vitamin D levels in pregnant women and the implementation of appropriate interventions are recommended to enhance pregnancy outcomes.

## Data Availability

The original contributions presented in the study are included in the article/**Supplementary Material**, further inquiries can be directed to the corresponding authors.
