# Supplementary material for: The effect of vitamin D deficiency during pregnancy on adverse birth outcomes in neonates: a systematic review and meta-analysis
Source: Front Pediatr. 2024 May 14;12:1399615. doi: 10.3389/fped.2024.1399615 (PMC11130944; doi:10.3389/fped.2024.1399615)
Supplement: Supplementary file 1 [file Datasheet1.pdf]

Supplementary Material

1 Supplementary Tables

1.1 Supplementary Tables

Supplementary Table 1 Pubmed search strategy

NO.	Search query	Results
1	Search:"Vitamin D Deficiency"[Mesh] Sort by Most Recent	32671
2	Search:(((Vitamin D Deficiency[Title/Abstract]) OR (Deficiency, Vitamin D[Title/Abstract])) OR (Deficiencies, Vitamin D[Title/Abstract])) OR (Vitamin D Deficiencies[Title/Abstract])	16880
3	(#1) OR (#2)	39023
4	Search:"Pregnancy"[Mesh] Sort by Most Recent	1008895
5	Search:((pregnancy[Title/Abstract]) OR (Pregnancies[Title/Abstract])) OR (Gestation[Title/Abstract])	589262
6	(#4) OR (#5)	1144397
7	Search:"Infant, Newborn"[Mesh] Sort by Most Recent	674932
8	Search:((((((Infant, Newborn[Title/Abstract]) OR (Newborn Infant[Title/Abstract])) OR (Newborn Infants[Title/Abstract])) OR (Newborns[Title/Abstract])) OR (Newborn[Title/Abstract])) OR	287626

	(neonate[Title/Abstract])) OR (Neonates[Title/Abstract])	
9	(#7) OR (#8)	780289
10	((#3) AND (#6)) AND (#9)	890

Supplementary Table 2 Basic characteristics of the included literature

References	Country	Single /Multi(center)	Design	Study cycle	Gestational period(tri mester)	25-hydroxyvitamin D detection	Sample size	
							Experimental	Control
J Zhou, 2014	Guangzhou, China	Single	prospective cohort study	2010.09-2011.08	Second	Electrochemical luminescence immunoassay	370	637
A Rodriguez, 2014	Spain	Multi-	prospective cohort study	2003-2008	Second	-	400	1104
Yuan-Hua Chen, 2015	Anhui,China	Multi-	cohort study	2009.01-2009.12	whole pregnancy	Electrochemical luminescence immunoassay	1405	964
Yi Lin Ong，2016	Singapore	Multi-	cohort study	-	Second	Liquid chromatography-tandemmass spectrometry	120	549
Chui Ling Lee，2017	Malaysia	Single	cohort study	2013.08-2015.08	Third	Liquid chromatography-tandem mass spectrometry	412	42
Yuan-Hua Chen，2018	Anhui, China	Single	prospective cohort study	2009.01-2009.12	whole pregnancy	Electrochemical luminescence immunoassay	1397	941
Li Yu，2019	Guangdong, China	Single	Retrospective cohort study	2016.01-2019.04	whole pregnancy	Electrochemical luminescence immunoassay	4051	2583
Isabelle Monier，2019	France and Belgium	Multi-	prospective cohort study	2012.04-2014.07	First	Electrochemical luminescence immunoassay	1268	677

Meng Ni，2021	Shanghai, China	Single	Retrospective cohort study	2015.01-2016.12	First	Electrochemical luminescence immunoassay	15696	717
Bo Chen, BD，2021	Anhui,China	Single	cohort study	2019.01-2020.06	whole pregnancy	Electrochemical luminescence immunoassay	2565	42
Natalia Perez-Ferre, 2012	Singapore	Single	cohort study	2010.06-2010.09	Second	Electrochemical luminescence immunoassay	157	25
Alison D.Gernand, 2014	America	Multi-	cohort study	1991-1995	Second	Liquid chromatography-tandem mass spectrometry	290	254
Kawakib Hussein Noamam, 2021	Baghdad, Iraq	Single	prospective cohort study	2017.04-2018.03	First	Liquid chromatography-tandem mass spectrometry	24	45

Supplementary Table 3 NOS score of studies included in this meta-analysis

References	Selection				Comparability	Exposure			Total
	①	②	③	④		①	②	③	
J Zhou, 2014	★	★	★	★	★	★	★	★	8
A Rodriguez, 2014	★	★	★	★	★★	★	★	★	9
Yuan-Hua Chen, 2015	★	★	★	★	★	★	★	★	8
Yi Lin Ong, 2016	★	★	★	★	★★	★	★		8
Chui Ling Lee, 2017	★	★	★	★	★	★	★		7
Yuan-Hua Chen, 2018	★	★	★	★	★★	★	★	★	9
Li Yu, 2019	★	★	★	★	★★	★	★		8
Isabelle Monier, 2019	★	★	★	★	★★	★	★	★	9
Meng Ni, 2021	★	★	★	★	★★	★	★		8
Bo Chen, BD, 2021	★	★	★	★	★	★	★	★	8
Natalia Perez-Ferre, MD, 2012	★	★	★	★	★	★	★	★	8
Alison D. Gernand, 2014	★	★	★	★	★★	★	★	★	9
Kawakib Hussein Noamam, 2021	★	★	★	★	★	★		★	7
